# Amyloid-like ribbons of amelogenins in enamel mineralization

**DOI:** 10.1038/srep23105

**Published:** 2016-03-24

**Authors:** Karina M. M. Carneiro, Halei Zhai, Li Zhu, Jeremy A. Horst, Melody Sitlin, Mychi Nguyen, Martin Wagner, Cheryl Simpliciano, Melissa Milder, Chun-Long Chen, Paul Ashby, Johan Bonde, Wu Li, Stefan Habelitz

**Affiliations:** 1 Department of Preventive and Restorative Dental Sciences, School of Dentistry, University of California, San Francisco, CA 94143, USA; 2 Department of Orofacial Sciences, School of Dentistry, University of California, San Francisco, CA 94143, USA; 3 Department of Biochemistry and Biophysics, School of Medicine, University of California, San Francisco, CA 94158, USA; 4 Bruker Nano Surfaces Division, 112 Robin Hill Road, Santa Barbara, CA 93117, USA; 5 The Molecular Foundry, Lawrence Berkeley National Laboratory, One Cyclotron Road, Building 67, Berkeley, CA 94720, USA; 6 Pacific Northwest National Laboratory, PO Box 999, MSIN: K4-18, Richland, WA 99352, USA; 7 Division of Pure and Applied Biochemistry, Center for Applied Life Sciences, Lund University, P.O. Box 124, SE-221 00, Lund, Sweden

## Abstract

Enamel, the outermost layer of teeth, is an acellular mineralized tissue that cannot regenerate; the mature tissue is composed of high aspect ratio apatite nanocrystals organized into rods and inter-rod regions. Amelogenin constitutes 90% of the protein matrix in developing enamel and plays a central role in guiding the hierarchical organization of apatite crystals observed in mature enamel. To date, a convincing link between amelogenin supramolecular structures and mature enamel has yet to be described, in part because the protein matrix is degraded during tissue maturation. Here we show compelling evidence that amelogenin self-assembles into an amyloid-like structure *in vitro* and *in vivo*. We show that enamel matrices stain positive for amyloids and we identify a specific region within amelogenin that self-assembles into β-sheets. We propose that amelogenin nanoribbons template the growth of apatite mineral in human enamel. This is a paradigm shift from the current model of enamel development.

Amyloids, a class of proteins that spontaneously self-assemble into fibrous cross-β aggregates, have been associated with the pathology of amyloidosis and neurodegeneration^1^. Recently, however, non-toxic amyloids with functional roles in biosynthetic pathways were discovered, challenging our current views on the sole role of amyloids in mammals to be cytotoxic^2^,^3^,^4^. Here we show that amelogenin, the principal constituent of the developing enamel matrix, self-assembles into one-dimensional β-aggregates characteristic of amyloids, and we propose that these provide structural support in guiding mineralization during tooth enamel development. We identify the determinant region of self-assembly, and demonstrate evidence of amyloid-like structure in recombinant human amelogenin assembly *in vitro* and in developing murine tooth *in vivo*.

Enamel is the outermost layer of a tooth and is the hardest and most mineralized tissue in vertebrates. The mature tissue is a highly organized structure comprised of single-crystalline apatite fibers of about 50 nm in diameter and several hundreds of microns in length^5^. It is generally accepted that this crystal morphology and alignment are the result of a protein-guided uniaxial growth process. The exact mechanism guiding this process remains undetermined^6^, obfuscated by the rapid degradation of the underlying protein matrix during tissue maturation. The role of self-assembly of enamel matrix proteins, in particular amelogenin, has been widely recognized as a crucial factor in controlling enamel structural development. While evidence for filamentous protein nanostructures and the presence of β-sheets in developing enamel have been demonstrated by transmission electron microscopy (TEM) and x-ray diffraction (XRD) respectively^7^,^8^, the premise that amyloid fibers template the oriented growth of apatite in enamel has not been evaluated.

Human full-length amelogenin (H175) consists of 175 amino acid residues and is predominantly hydrophobic, apart from its C-terminus and a phosphorylated serine residue (Ser16). Due to its amphiphilic nature, the full-length protein aggregates in aqueous conditions *in vitro* with the main morphology observed being nanospheres^9^,^10^,^11^. Previous studies on amelogenin self-assembly paid little attention to the solution ionic strength and composition, and the fact that quaternary structure formation by the assembly of large macromolecules may require long periods of time (hours or days) and not seconds or minutes. Calcium and phosphate ions are present during enamel formation and their concentration gradually increases during enamel maturation^12^. Recently, we discovered that the recombinant human full-length amelogenin protein (rH174) and its matrix metalloproteinase-20 (MMP20) cleavage product, rH146, self-assemble into nanoribbons *in vitro*^13^,^14^,^15^,^16^. Amelogenin ribbon formation requires the presence of both calcium and phosphate ions, suggesting that ion bridges develop and drive the self-assembly process. The rH174 ribbons measure 3.4 nm in height, 16.7 nm in width and grow to tens of microns in length over a period of days^15^,^16^. Fourier transform infrared (FTIR) spectroscopy analysis showed vibrations associated with random-coil structure and β-sheet motif when amelogenin assembled into nanospheres and nanoribbons, respectively. The nanoribbons had 4.7 Å spacing when analyzed by synchrotron-based x-ray diffraction (XRD), consistent with amyloid cross-β sheet quaternary structure^13^. Herein we describe a mechanism for amelogenin self-assembly into an amyloid-like structure *in vitro* and *in vivo*, on the basis of a specific region within the protein sequence capable of adopting a β-sheet configuration with antiparallel dimers that drive the subsequent ribbon assembly.

## Results and Discussion

**Figure 1:**
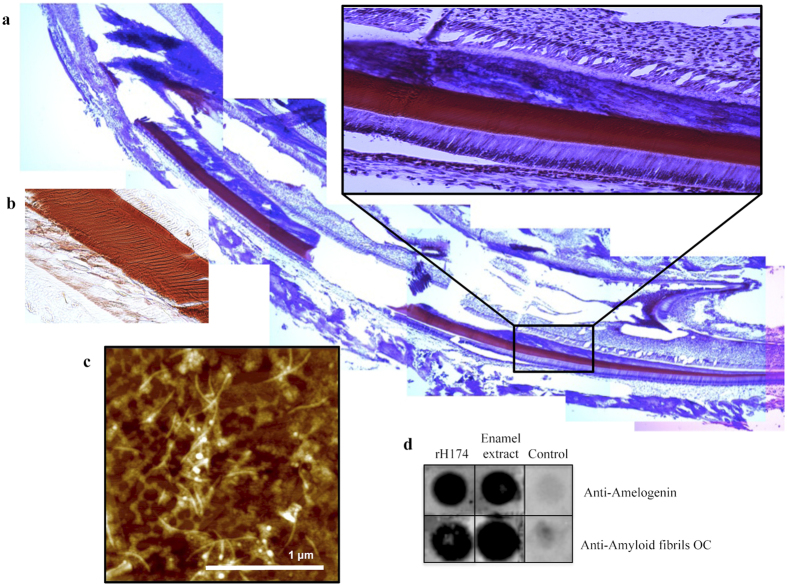
*In vivo* studies indicate the presence of amyloid-like assembly in developing enamel. (**a**) Incisor of KLK4 null mouse stained with Congo Red and hematoxylin; intense red stain along the complete length of incisor enamel from secretory stage at the cervical loop (right) to the end of maturation stage in the erupted incisor is observed. Higher magnification shows ameloblasts and odontoblasts with dentin layer adjacent to strongly red stained enamel layer. (**b**) Incisor from KLK4-null mouse stained positively with Congo Red - position maturation stage with enamel rods visible. (**c**) AFM analysis of enamel matrix extracted from un-erupted porcine teeth shows fibrillar structures similar to recombinant amelogenin ribbons *in vitro*. (**d**) Dot blot analysis of (1) rH174, (2) porcine enamel extract and (3) bovine serum albumin (BSA) with anti-amelogenin and anti-amyloid OC antibodies. rH174 and porcine enamel extract samples stain positive with both antibodies, suggesting that amelogenin and amyloids are present.

Developing enamel matrix is composed of equal parts by mass of water, minerals and proteins. Amelogenin is the main protein present, and its C-terminus is processed fairly rapidly by MMP20 during the secretory stage. Further processing occurs during the maturation stage, after the entire thickness of enamel has been obtained; kallikrein-4 (KLK4) is secreted and removes the matrix almost completely, providing space for lateral growth of apatite crystals^17^,^18^. Using mice that lack KLK4 and thus the ability to process amelogenin, temporarily provides a more stable matrix and a feasible approach to analyze its structure *in vivo*. We used Congo Red stain on untreated mineralized mandibular sections of KLK4 knockout (KO) mice and compared to wild-type (WT) mice to test for the presence of β-sheets. Congo Red strongly stained the enamel in all teeth, incisors and molars, in the KLK4-KO specimen (Fig. 1a,b). An intense red stain was observed from the secretory stage through the maturation stage and into the erupted portion of the tooth, demonstrating that inhibition of KLK4 processing can preserve protein structures in the enamel matrix. Staining of sectioned non-demineralized WT specimens was challenging, but the few remaining segments of enamel in WT incisor also suggested the presence of amyloid in developing enamel (Fig. S1). In addition, we extracted porcine developing enamel and tested for the presence of amyloids. When visualized by atomic force microscopy (Fig. 1c), the extract was composed of a large amount of nanoribbons that had a striking resemblance to recombinant human amelogenin nanoribbons previously reported by our group^14^. The enamel extract tested positive for amelogenin when analyzed by gel electrophoresis and western blot (Fig. S2). Porcine enamel extract tested positive for amyloids as indicated by Thioflavin T (ThT) fluorescence as well as when analyzed by dot blot with amyloid-specific antibodies such as OC and 4G8 (Figs 1d and S3).

**Figure 2:**
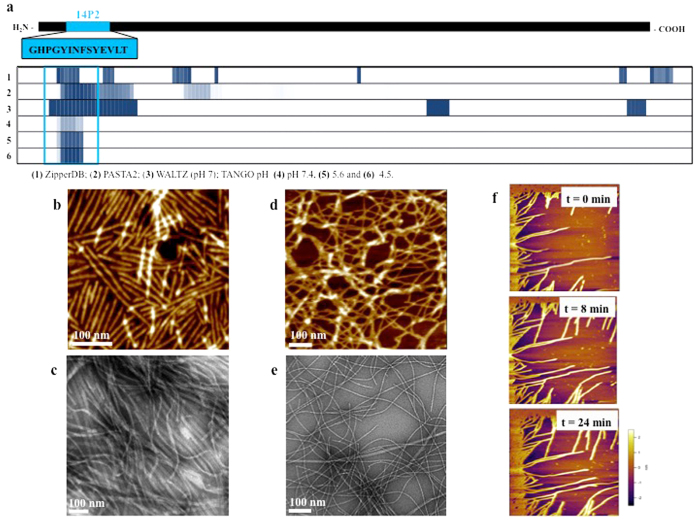
β-sheet aggregation predictions and self-assembly of rH174 and 14P2. (**a**) Secondary structure analyses shows up to seven domains within amelogenin sequence to have a high propensity for β-sheet aggregation and possible amyloid formation. In particular a region near the N-terminus (14P2) showed high propensity for these structures for all analysis methods used: (1) ZipperDB, (2) PASTA2, (3) WALTZ (pH 7) and TANGO at pH (4) 7.4, (5) 5.6 and (6) 4.5. TANGO algorithm predicts an increase for amyloid propensity with lower pH values. Intensity of the blue color is proportional to relative propensity for β-sheet aggregation. (**b**) Recombinant human amelogenin (rH174) demonstrates pH-dependent self-assembly into nanoribbons in the presence of calcium (33.4 mM) and phosphate (20.9 mM) ions as shown by AFM and (**c**) TEM of nanoribbon bundles formed after 7 days of incubation. (**d**) AFM analysis of 14P2 assembling into ribbons in the presence of calcium (3.3 mM) and phosphate (2.1 mM) ions. The ribbons do not align in a side-to-side manner as rH174 ribbons do. (**e**) TEM of 14P2 ribbons with negative stain measure about 6.6 nm in width and are several microns long. (**f**) Real-time images of 14P2 self-assembling into ribbons in fluid conditions on mica surfaces at calculated rates of 5 nm/min.

Stimulated by the data suggesting amyloid-like structures in the matrices of murine and porcine enamel, we employed seven bioinformatics amyloid or fibrillation prediction methods on the amelogenin amino acid sequence. A cluster of highest β-aggregation scores for the region from threonine-12 to leucine-20 was predicted by all methods (Figs 2a and S4). These methods apply a sliding-window analysis, and differ in analytic parameters input into the machine learning algorithms. For example, TANGO compares estimates of desolvation energy, electrostatic interactions, and secondary structure propensity for the query peptide to 179 known amyloid peptides without atomic modeling^19^; while ZipperDB estimates interface and enthalpic properties after generating an atomic model by mapping the sequence to one amyloid peptide structure^20^. The TANGO analysis method calculates decreasing pH to be a strong predictor of amyloid propensity for the region near the N-terminus of the protein.

In parallel we synthesized a tiling library of peptides from the amelogenin sequence, each comprised of 14 residues with a 7-amino acid overlap, and measured their intermolecular force using dynamic force spectrometry (DFS) with an atomic force microscope (AFM). Our results identified a peptide 14P2 (residues 8–21, GHPGYINFSYEVLT) that had extremely high self-binding affinity, ranging from 10.70–17.36 k_b_T at different ionic conditions (Table S1). These values are slightly lower than those obtained for amyloid forming peptides Aβ40 and Aβ42 (27.3 and 27.7 k_b_T respectively), associated with Alzheimer’s disease^21^,^22^. The peptide 14P2 is located near the N-terminus of amelogenin and falls exactly within the region predicted to form β-sheet aggregates by our models (Fig. 2a).

Previous studies have reported that the N-terminus of amelogenin (residues 1–42) is required for protein-protein interactions and that mutations to amino acids in this region may lead to altered self-assembly dynamics^23^. As a control, we expressed full-length amelogenin with the 14P2 peptide region deleted from the amino acid sequence of the protein, to synthesize 14P2-del-rH174. Under the same reaction conditions as the ones previously described, we observed that 14P2-del-rH174 did not assemble into nanoribbons in the presence of calcium and phosphate ions even after two months of incubation (Fig. S5). Thus, 14P2 amino acid sequence in rH174 is essential for the one-dimensional assembly of the full-length protein into ribbons, due to its ability to form β-sheets that drive self-assembly into nanoribbons.

The self-assembly of 14P2 into supramolecular structures was investigated using similar conditions to those previously used for rH174 assembly into nanoribbons (14). The self-assembly of 14P2 in aqueous solutions containing calcium and phosphate ions at pH 4.5–5.5 was monitored over several days, using circular dichroism (CD), atomic force microscopy (AFM) and transmission electron microscopy (TEM). Similar to the full-length protein, the peptide assembled into ribbons of several microns in length and 3.0 (±0.9) nm in height. Nanoribbon length was dependent on sample concentration, with shorter ribbons observed at higher peptide concentrations (Fig. S6). The assembly of 14P2 occurred at much faster rates (a few hours compared to several days) than the full-length protein (Fig. 2b,c). In addition, no parallel alignment of the peptide ribbons was observed, in contrast to the full-length protein (Fig. 2d). 14P2 assembly into long fibers was also analyzed by TEM using a negative stain, with ribbons measuring 6.6 (±3.4) nm in width (Fig. 2e). In unstained samples however, fewer ribbons were observed (Fig. S7). Ribbon formation of 14P2 was followed in real-time by *in situ* AFM (Fig. 2f), where a dense carpet of ribbons developed within seconds on mica substrates and only in areas surrounding the moving AFM cantilever ribbon formation was blocked. Over time (24 minutes) the ribbons elongated at a calculated speed of 5 nm/min. When either calcium or phosphate ions, or both, were left out of the assembly solution, fiber formation was inhibited or strongly restricted, with very few fibers observed (Fig. S8). The dependence on calcium and phosphate ions for nanoribbon assembly is identical to the behavior of the full-length protein rH174.

**Figure 3:**
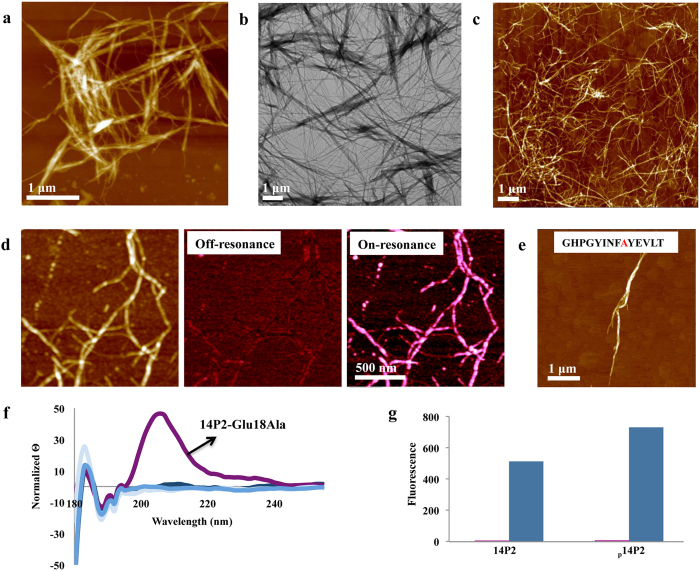
Phosphorylated 14P2 (_p_14P2) self-assembles into nanoribbons as a function of calcium and phosphate ions. (**a**) AFM of _p_14P2 nanoribbons in the presence of calcium (3.3 mM) and phosphate (2.1 mM) ions. These ribbons had a higher tendency to bundle into micron-sized aggregates than the ones from 14P2. (**b**) TEM of _p_14P2 nanoribbons bundles unstained. (**c**) AFM of _p_14P2 nanoribbons in absence of phosphate ions. (**d**) AFM-based s-SNOM measurements of _p_14P2 nanoribbons in absence of phosphate ions: topography (left), off-resonance infrared near-field absorption at 1028.5 cm^−1^ (middle) and on-resonant absorption at 1097 cm^−1^ (right). (**e**) Mutation of Ser16Ala restricted supramolecular development and resulted in few ribbons as visualized by AFM, indicating the importance of serine-16 for ribbon formation. (**f**) Circular dichroism of 14P2 and of its modifications (Ser16Ala, Glu18Ala and Glu18Gln). In all cases, dichroic peaks indicative of β-sheets are observed. For 14P2-Glu18Gln, a dichroic peak indicative of an α-helix is also present. (**g**) 14P2 and _p_14P2 show fluorescence when Thioflavin T (blue) is added, suggesting the presence of amyloids.

Since the native amelogenin H175 is phosphorylated at serine-16 (Ser16), we synthesized a peptide (_p_14P2) using phosphorylated serine. Similar to 14P2 and rH174, _p_14P2 assembled into long ribbons in the presence of calcium and phosphate ions. The _p_14P2 ribbons measured 5.9 (±2.0) nm in height as determined by AFM (Fig. 3a), and 10.7 (±2.9) nm in width as determined by TEM with negative stained specimens (Fig. S9). These ribbons had a higher tendency to intertwine than 14P2, forming dense bundles measuring up to 40 nm in height. Agglomerated ribbons were also observed by TEM even when the sample was not stained (Fig. 3b). The darker appearance of the _p_14P2 ribbons when compared to the non-stained 14P2 TEM sample is likely due to the increased incorporation of electron-dense phosphate groups into the ribbons. Importantly and in contrast to 14P2 assembly, fiber formation readily occurred in the absence of phosphate ions for _p_14P2 (Fig. 3c), illustrating that phosphorylation of serine-16 can substitute for the addition of inorganic phosphate and may thus play a vital role in amelogenin self-assembly. Phosphorylation of peptides has been used to improve their long-range assembly^24^. The addition of calcium was still required to produce stable peptide ribbons of _p_14P2; in its absence peptide ribbons became scarse and very few were observed (Fig. S10). This behavior points to the essential role of calcium ions in the intermolecular interactions required for ribbon formation. The spatial distribution of phosphate within 14P2 assembly in the absence of inorganic phosphate with <20 nm spatial resolution was mapped using scattering-type scanning near-field optical microscopy (s-SNOM) at infrared frequencies. This technique is ideal to simultaneously obtain topography and chemical content information of materials^25^. Figure 3d shows topography (left) and near-field absorption images off- (center) and on-resonance (right) with the phosphate absorption line. There is little absorption contrast off-resonance with the phosphate absorption line while the nanoribbons become clearly visible for on-resonance imaging, demonstrating the presence of phosphate groups throughout the assembly.

In order to assess the role of specific amino acids and their interactions with mineral ions in the supramolecular assembly of 14P2, residues of peptide 14P2 were mutated. We tested the effect of replacing serine with another residue. When serine-16 was mutated to alanine (Ser16Ala) the peptide did not readily assemble, and only rarely fibers, usually as bundles, were observed by AFM (Fig. 3e). These results further emphasize a central role of serine-16 in the assembly of 14P2 into nanofibers. A second set of 14P2 peptide modifications was designed to investigate the specificity of interactions with calcium ions. Two point mutations to charged amino acid glutamate-18 were introduced: modification Glu18Gln decreases calcium affinity and probes for the role of the negative charge, and the modified peptide Glu18Ala lowers the potential for electrostatic interactions. The 14P2-Glu18Gln peptide formed helical fibers, as observed by AFM (Fig. S11). Circular dichroism (CD) of this peptide showed dichroic peaks indicative of β-sheets similar to the original peptide (Fig. 3f). When glutamate-18 was replaced with alanine (Glu18Ala), ribbons no longer developed, but instead thin sheets were observed by AFM and TEM (Fig. S12). In this case, CD analysis showed an extra peak indicating the appearance of a random coil structure (Fig. 3f). These mutations indicate that glutamate-18 plays a role in protein structure, but is not necessary to drive long-range assembly.

**Figure 4:**
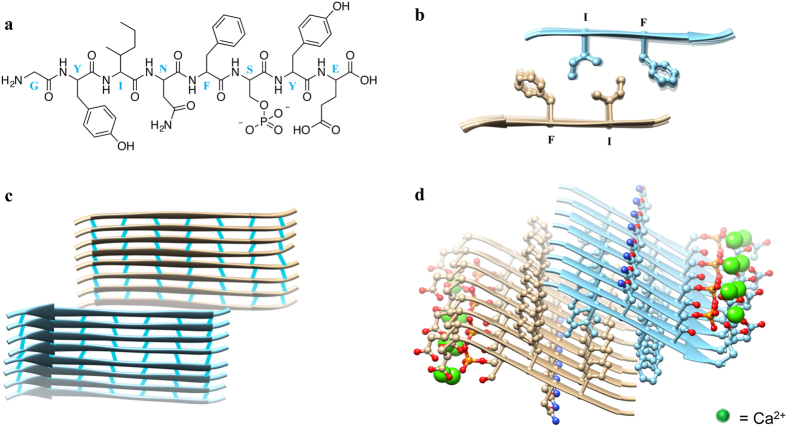
An atomic structural model of the phosphorylated peptide GYINFS_p_YE was developed using the steric Zipper data bank. (**a**) Chemical structure of the peptide. (**b**) Antiparallel dimers develop through hydrophobic interactions between isoleucine and phenylalanine residues, forming a dry interface. (**c**) Dimers elongate into steric zippers through hydrogen bonds between the backbones of neighboring peptides. (**d**) Self-assembly into ribbons is facilitated by alignment of dimers through calcium ions along the outer/hydrated layer by bridging with glutamate and phosphoserine residues. Atomic structural model of peptide assembly is shown for the sequence of _p_14P2, based on the *Saccharomyces cerevisiae* sup35 protein and fits with the prion cross-β amyloid architecture^53^.

14P2 is of similar length and has similar assembly behavior as other amyloid-forming peptides^26^,^27^. Addition of Thioflavin T to 14P2 and _p_14P2 solutions produced fluorescence characteristic of β-sheet aggregates and amyloid fibers (Fig. 3g)^28^. It has been previously reported that phosphorylation and the presence of calcium ions in solution increase the aggregation kinetics in amyloid beta (Aβ) peptides^29^,^30^. To date, there is substantial evidence in the literature suggesting the occurrence of fibrillar assemblies with possible amyloid characteristics in developing enamel^13^,^31^,^32^,^33^,^34^,^35^,^36^,^37^,^38^,^39^. An atomic structure of amelogenin would be highly beneficial in predicting the presence of β-sheets in its secondary and tertiary structures and in modeling its long-range assembly behavior. Nonlocal atomistic structural determinants to inform amelogenin structure have not been resolved, and no template is available for structure-based modeling (Protein DataBank, accessed 4/16/2015). We attempted *de novo* structure prediction for amelogenin using multiple methods. Regions with consistent and compact structural motifs were found only with QUARK^40^, the most accurate and sensitive method^41^. For the GYINFSYE region formation of β-sheets was consistently predicted. We then used ZipperDB to predict the optimal interface residues (GYINFS), and draft a quaternary assembly based on the sup35 NNQQNY template. We modeled the remaining residues and phosphoserine manually in CHIMERA^42^. Calcium ions were initially docked with RMR6^43^, and after noticing a pattern were placed to match the hydroxyapatite structure. The resulting model is shown in Fig. 4, and depicts a steric zipper with a hydrophobic interface. Figure 4a shows the model for the peptide sequence GYINF_p_SYE in the extended β-strand configuration with hydrophobic residues facing one side and polar residues facing the opposite side. Two peptides form an antiparallel dimer through hydrophobic interactions between isoleucine-13 and phenylalanine-15 (Fig. 4b). The dimers extend through a hydrogen-bond network in the backbone of adjacent peptides and a central calcium binding site comprised of two glutamate-18 residues and a phosphate group from phosphorylated serine (Fig. 4c,d), illustrating the role of calcium and phosphate for self-assembly. An additional hydrogen bond site can be found between asparagine-14 residues of adjacent peptides.

We next investigated the presence of amyloid character in the full-length amelogenin assemblies *in vitro*. Dot blot staining was used with antibody OC, well known for its non-sequence dependent recognition of amyloid fibrils and non-binding to random-coil monomers or pre-fibrillar oligomers^44^,^45^. Amelogenin rH174 tested positive for both amelogenin and amyloid staining in dot-blot analysis (Fig. 1d). Amyloid assembly kinetics can be increased through a seeding mechanism, where a small amount of aggregates rich in β-sheets stimulate the assembly of the native protein. In order to probe whether this mechanism may occur with amelogenin, we used 14P2 monomers (at a ratio of 10:1 rH174:14P2) as seeds to ribbons formation. Indeed, the assembly occurred at a much faster rate than the assembly of the protein alone, similarly to the seeding mechanism observed in amyloid fiber formation. 14P2 already assembles at a faster rate than rH174, thus we hypothesize that the monomers of 14P2 form β-sheet rich aggregates that act as seeds for the assembly of rH174 (Fig. S13).

**Figure 5:**
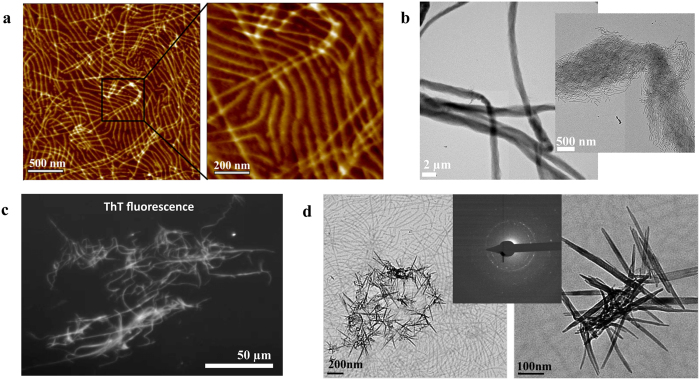
Self-assembly and mineralization of rH174-(+9). (**a**) Assembly of rH174-(+9) into highly aligned ribbons in the presence of calcium (33.4 mM) and phosphate (20.9 mM) ions at pH 6.5. The peak-to-peak distance between ribbons is 69.1 ± 7.8 nm. (**b**) TEM of large bundles composed of aligned rH174-(+9) nanoribbons in the presence of magnesium and phosphate ions. (**c**) rH174-(+9) bundles shown in (**b**), show fluorescence after staining with Thioflavin T, suggesting the presence of amyloids. (**d**) Mineral deposition on aligned rH174-(+9) ribbons visualized by TEM at pH 6.5. Small angle electron diffraction (SAED) of the minerals shows rings indicative of a crystalline structure (inset).

Filamentous protein assemblies have been previously reported in the developing enamel matrix and were associated with amelogenin as the main constituent^36^. We therefore propose that amelogenin nanoribbons act as a guide for mineral formation and template the growth of apatite crystals along its backbone. Apatite mineral develops during the secretory stage with high aspect ratio, about 10–20 nm wide and only 3–4 nm thick^46^. These elongated crystals are aligned in parallel and continue to grow in synchrony with the ameloblast migratory pathway. Crystal orientation is dependent on whether the protein matrix is secreted from the distal or the proximal end of Tomes’ process of ameloblasts; these different secretion sites are responsible for the oblique angle observed between apatite crystals in enamel rod and inter-rod regions^45^. A clear association of mineralization with the structure of amelogenin rH174 has not been described which may be by design, as in biological mineralization post-translational modification to a protein is commonly required for conversion into a molecule that promotes mineralization^14^. The C-terminus of amelogenin is hydrophilic and comprised of numerous charged residues, suggesting it plays a major role in interactions with mineral ions. It has been previously shown that the C-terminus has been associated with HAP binding. In an attempt to induce mineralization of amelogenin nanoribbons, we modified its C-terminus by extending it with a triple repeat of amelogenin sequence KTKR^47^,^48^, and tested the effect of additional electrostatic charges on self-assembly and mineralization. The resulting rH174-(+9) protein was exposed to the same self-assembly protocol as described above and also assembled into ribbons that were organized in a parallel fashion, but with extremely regular distances of about 69 nm between ribbons when immobilized on glass surfaces (Fig. 5a). These ribbons had significantly increased width (22.9 nm) compared to the rH174 protein (16.7 nm), supporting our assembly model that the C-terminus of the protein is oriented toward the outer edge of the nanoribbons^14^. Using AFM-based infrared nano-imaging (s-SNOM), the location of the inorganic phosphate was associated with the protein nanoribbons as indicated by a strong increase in the infrared absorption signal at a wavenumber around 1100 cm^−1^ (Fig. S14). Modified amelogenin rH174-(+9) also assembled into ribbons when magnesium substituted calcium ions. In the presence of magnesium ions, ribbons had an even higher tendency to self-align and to aggregate into large bundles of parallel ribbons. These rods are hundreds of microns in length, around 2 μm in width, and were easily observed by TEM without staining (Fig. 5b) and even by optical microscopy (Fig. S15). Staining of the rH174-(+9) ribbons with ThT resulted in fluorescence of the amelogenin ribbons (Fig. 5c), further supporting the presence of an amyloid character in these protein structures. At times, elongated minerals developed during the self-assembly of rH174-(+9) at pH 6.5 as observed by TEM (Fig. 5d). We performed small angle electron diffraction (SAED) to confirm that the mineral at this stage is crystalline (Fig. 5d, inset). Micrometer-sized apatite crystals also developed randomly in the solution (Fig. S16). Guided crystal growth in association with amyloid ribbons is currently being investigated.

## Conclusions

The underlying mechanism guiding enamel mineralization and the formation of highly oriented apatite nanofibers is still not well understood. Our results indicate that the developing enamel matrix is not only comprised of proteins adapting a β-sheet configuration, but also capable of developing one-dimensional assemblies with amyloid characteristics. These filaments are 3–4 nm in thickness and exhibit very similar characteristics to recombinant amelogenin nanoribbons previously reported^14^. A region near the N-terminus of the protein, 14P2, is crucial for amelogenin self-assembly into nanoribbons through β-sheet interactions *in vitro*. We have demonstrated that phosphorylation of serine-16 in 14P2, as is observed *in vivo*, alleviates the need for inorganic phosphate for ribbon assembly and may thus be critical to ribbon assembly *in vivo*. Our data suggests that dimers connect directly through the phosphorylated sites and glutamate residues through divalent calcium ions, promoting ribbon elongation and the development of a β-sheet structure or amyloid-like fiber; this ion bridge involves the interaction of the calcium ions with phosphate groups in the protein and illustrates the pivotal role of phosphorylation in assembly.

To date, very few examples of non-disease causing, naturally occurring, functional amyloids in mammals have been discovered^2^,^3^,^4^,^49^. Based on the experiments described herein, we conclude that full-length amelogenin, a unique protein with little sequence identity to any other mammalian protein and the main component in developing enamel, adopts an amyloid-like motif *in vitro* and *in vivo*. This is the first time that an amyloid motif has been linked with biomineralization; this model may provide an alternative molecular mechanism in enamel development.

## Methods

### SI Materials and Methods

#### Mouse tooth sample fixation

Heads from wild-type (WT) mice and KLK4 knockout (KO) mice, obtained from the Simmer laboratory, in accordance with the approved guidelines for animal care at the University of Michigan, were immediately fixed in 10% neutral buffered formalin and stored in phosphate buffer saline (PBS). Mandibular and maxillary specimens were dissected after additional fixation with 4% paraformaldehyde (PFA) in 0.1 M cacodylate buffer for two days, then rinsed with 0.1 M cacodylate buffer and fixed again overnight in 4% PFA (in 0.1 M cacodylate buffer). After the second fixation, they were washed in 0.1 M cacodylate buffer and then alcohol dehydrated. Samples were then stored in 100% ethanol, before embedding in paraffin wax and sectioning with microtome at about 80 µm thickness.

#### Histological and immunohisto-chemical identifications of amyloid

The histochemical and immuno-histochemical stains include H&E, amelogenin, MMP20 and KLK4. Amyloid staining include Congo Red plus polarized microscope and Thioflavin T (ThT, binds to β-sheet rich structures of amyloid aggregates)^50^. We also used the amyloid conformation-specific and Aβ42 sequence-specific antibodies, OC (EMD Millipore) and 4G8 (BioLegend), for immunohisto-chemistry to identify the amyloid in the tissues^44^.

#### Dot blot

Recombinant amelogenin proteins (500 ng/μL) samples were incubated at 37 °C in buffer containing 50 mM Tris-HCl pH 7.5, 10 mM CaCl_2_ and 150 mM NaCl for one hour. BSA (500 ng/μL) was used as a negative control. 5 μL of each sample were spotted in triplicate onto nitrocellulose membrane and dried at room temperature under vacuum. The membrane was then briefly rinsed in TBS (tris-buffer saline, pH 7.4) and was incubated overnight at 4 °C in 5% skim milk. The primary antibody anti-rH174 or anti-amyloid fibrils OC (EMD Millipore) was then added at 1:1000 dilution in TBST (TBS-tween) and the membrane was incubated for one hour. Next, the membrane was washed three times and secondary antibody conjugated with HRP conjugated anti-rabbit IgG (Bio-Rad) was used at 1:1000 dilution in TBST. One hour incubation was followed by three washes. Amersham ECL Western Blotting Detection Reagent was used to detect the signal.

#### Modeling

The cross-β sheet structure was modeled by threading the amelogenin sequence through the NNQQNY from the sup35 prion protein of *Saccharomyces cerevisiae* structure using ZipperDB. The sequence GYINFS received the most favorable score. The resulting model was extended to include the GYINFSYE sequence using the “Build Structures” tool in UCSF Chimera. The model was then minimized using KobaMin. Every other serine was mutated to phosphoserine, again with UCSF Chimera. Calcium ions were docked using RMR6^43^. It was noticed that the calcium-phosphate pattern matched the organization of hydroxyapatite, so further phosphate and calcium ions were added using custom scripts based on theoretical geometric pattern of hydroxyapatite.

#### Dynamic Force Spectroscopy (DFS)

The levers D or E of SiN_4_ AFM tips (MSCT, Bruker AFM Probes, Santa Barbara, CA) were used in all DFS experiments. Bare MSCT tips were first cleaned with a plasma cleaner for 20 s and immediately coated with 4 nm Cr and 20 nm Au, respectively, by thermal evaporation methods. The tip cross-linking was performed following a previously reported method^51^. The functionalized AFM tip was gently engaged to the gold surface by tapping mode to protect the surface chemistry and then switched to contact mode. To avoid the heterogeneous modification of gold surface, a round walk program was applied to randomly sample the sites on the surface. The force distance and trigger force were set to 200 nm and 200 pN, respectively. The 14P2 linked AFM tip was approached to the gold chip surface at a constant rate of 500 nm/s; the tip dwelled for 2 s before being pulled away at five different velocities between 5 × 10^−8^ m/s and 3 × 10^−6^ m/s. 200 force curves were collected for each retreating velocity. Finally, the interaction energy was calculated based on previously developed multiple molecule models^21^.

#### Self-assembly

Protein and peptide samples were prepared in the same manner. The sample was dissolved in 500 μL of 1 mM HCl. Calcium and phosphate stock solutions were prepared using reagent grade chemicals CaCl_2_ (13.4 mM) and KH_2_PO_4_ (8.4 mM) and filtered (0.22 μm). Suspensions of 1 mL total volume containing 1 mg/mL of amelogenin or of peptide were prepared by adding KH_2_PO_4_ in one sample vial containing rH174 and CaCl_2_ in a second one containing the same amount of rH174. The two vials were combined, resulting in a final concentration of 3.3 mM CaCl_2_ and 2.1 mM KH_2_PO_4_. The pH of the resulting solution was adjusted to 4.5–5.5 or 6.5 (as required) by addition of 1 M KOH. Protein solutions were then concentrated by evaporation at 37 °C for around 3.5 hours (to a volume of 100 μL), samples were then incubated on a closed vial at 37 °C.

#### Atomic Force Microscopy (AFM) in ambient conditions

As previously described, 15 μL of sample were placed onto a glass slide and incubated for 1 hour on a wet cell for immobilization. Samples were then washed with 100 μL of autoclaved deionized water and immediately dried with compressed air. AFM measurements were performed in ambient conditions, using a Multimode III (Digital Instruments, Santa Barbara, CA, USA) and silicon cantilevers (OTESPA-R3, Bruker AFM probes, Santa Barbara, CA, USA). The amplitude set point was adjusted to 75% of the free amplitude value to decrease the damage to the protein superstructure. The images were analyzed using Nanoscope Analysis software (v1.40).

#### Atomic Force Microscopy (AFM) in fluid conditions

The AFM images were taken in fluid cells using a Veeco Multimode VIII and Bruker ultra-sharp AFM cantilevers (MSNL-10). The amplitude set point was adjusted to 60–70% of the free amplitude value to decrease the damage to the protein superstructure.

#### Transmission Electron Microscopy (TEM)

As previously described, 5 μL of sample were deposited on a glow discharged carbon-coated copper grid (Ted Pella, Redding, CA, USA) and incubated for 60 seconds. The sample was wicked, and washed two times with autoclaved deionized water and air dried. For negative staining, the samples were treated with 2% methylamine tungstate solution (NANO-W, Yaphank, NY, USA) after being washed with autoclaved deionized water. The grids were imaged with a FEI Tecnai 12 TEM (FEI company, Hillsboro, OR, USA) at 120 kV. Data were acquired with a 4 × 4 k Gatan Ultra Scan CCD camera (Gatan, Pleasanton, CA, USA).

#### Infrared nano-imaging (s-SNOM)

Infrared nano-imaging of the amelogenin proteins was performed using a commercial scattering scanning near-field optical microscope (s-SNOM), the Inspire with PeakForce Tapping (Bruker Nano Surfaces, Santa Barbara, CA, USA). In the AFM based instrument the tip is illuminated via a monochromatic, tunable, low-noise quantum cascade laser (Daylight Solutions, San Diego, CA, USA). The tip-scattered light is then analyzed in an asymmetric Michelson interferometer with a two-phase homodyne detection scheme to directly obtain the nanoscale infrared absorption with a tip-limited spatial resolution of 10–20 nm^52^. Metal-coated AFM tips (Nanoworld Arrow NCPT) were used in tapping-mode at a frequency and amplitude around 250 kHz and 40 nm, respectively. Background-free imaging was achieved via signal demodulation at higher harmonics of the tapping frequency; in this case the second (main text) and third (SI) harmonics were chosen.

#### Expression and purification of rH174

Recombinant human amelogenin (rH174) and its mutant (14P2-del-rH174) were expressed in BL21DE3 pLysS *Escherichia coli* and purified on C4 hydrophobic beads. The purity of the protein was above 95% as assessed by HPLC. rH174 and 14P2-del-rH174 lacks the first amino acid residue (Met1) compared to native human amelogenin sequences.

#### Expression and purification of rH174-(+9)

A nucleotide sequence encoding the peptide KTKRKTKRKTKR was fused to the 3′ end of the rH174 gene using PCR. The resulting construct, termed rH174-(+9), was cloned into a pET11 expression vector and expressed in *E. coli* BL21 (DE3) cells. The recombinant protein was purified using an acid/heat treatment method (47), followed by extensive dialysis against 0.05% acetic acid and lyophilization. The purified protein was assessed by SDS-PAGE to have purity above 95% and the molecular weight of the protein was verified using MALDI-TOF mass spectroscopy.

#### Study Approval on Animal Use

The described animal experiments were done according to the basic protocols reviewed and approved by the Institutional Animal Care and Use Committees (IACUC) at the University of Michigan and in an agreement with the U.S. Public Health Service Policy on Human Care and Use of Laboratory Animals.

## Additional Information

**How to cite this article**: Carneiro, K. M. M. *et al*. Amyloid-like ribbons of amelogenins in enamel mineralization. *Sci. Rep.* **6**, 23105; doi: 10.1038/srep23105 (2016).

## Supplementary Material

Supplementary Information
