# Supplementary material for: Amyloid-like ribbons of amelogenins in enamel mineralization
Source: Sci Rep. 2016 Mar 24;6:23105. doi: 10.1038/srep23105 (PMC4806362; doi:10.1038/srep23105)
Supplement: Supplementary Information [file srep23105-s1.pdf]

Amyloid-like ribbons of amelogenins in enamel mineralization.

Supplement

Authors: Karina M. M. Carneiro[a,1], Halei Zhai[b,1], Li Zhu[b], Jeremy A. Horst[b,c], Melody Sitlin[a], Mychi Nguyen[a], Martin Wagner[d], Cheryl Simpliciano[a], Melissa Milder[a], Chun-Long Chen[e], Paul Ashby[f], Johan Bonde[f], Wu Li[b], Stefan Habelitz[a]

Author affiliations: [a]Department of Preventive and Restorative Dental Sciences, School of Dentistry, University of California, San Francisco, CA 94143; [b]Department of Orofacial Sciences, School of Dentistry, University of California, San Francisco, CA 94143; [c]Department of Biochemistry and Biophysics, School of Medicine, University of California, San Francisco, CA 94158; [d]Bruker Nano Surfaces Division, 112 Robin Hill Road, Santa Barbara, CA 93117; [e] Pacific Northwest National Laboratory, Richland, WA 99352 [f]Department of Energy, Materials Science Division, Lawrence Berkeley National Laboratory, Berkeley, CA 94720; [f]Division of Pure and Applied Biochemistry, Center for Applied Life Sciences, Lund University, P.O. Box 124, SE-221 00, Lund, Sweden.

Supplementary Information:

Porcine enamel sample preparation: Porcine mandibular molars were obtained from the Animal Sharing Program at UCSF in compliance with applicable laws and regulations. The molars were dissected from 2- to 5-day old mice as previously described (1). 0.5 mg of porcine enamel matrix was homogenized in 0.5 mL of tissue homogenization buffer (250 mM sucrose, 20 mM Tris, 1 mM EDTA and 1 mM EGTA) and mixed with 1.1 mL of cold formic acid. The samples were incubated at room temperature with constant rotation for two hours and centrifuged at 13,000 g for 10 minutes to collect the supernatant. Samples were characterized by

atomic force microscopy (AFM). AFM samples were prepared by depositing 10 μL of sample on mica, immediately washing with 100 μL of deionized autoclaved water and drying with compressed air. The sample was imaged by tapping mode AFM.

Western blot: 0.5 mg of porcine enamel matrix was homogenized in 0.5 mL of tissue homogenization buffer (250 mM sucrose, 20 mM Tris, 1 mM EDTA and 1 mM EGTA) and mixed 0.5 mL of 2x SDS sample buffer. The samples were subjected to SDS-PAGE and were electrotransferred onto a membrane (Invitrogen). Polyclonal antibody for amelogenin was used for one hour at a dilution of 1:1000. Secondary antibody was HRP conjugated anti-rabbit IgG (Bio-Rad). The membrane was treated with ECL western blotting detection reagent for one minute and then exposed to Hyperfilm-ECL (Amersham).

Circular Dichroism (CD): CD spectra were obtained on a Jasco-715 CD spectrometer (JASCO Corporation, Japan) in a 1 mm cell at 25°C. Spectra were recorded under a continuous purge of 15-20 L/min of nitrogen using the Spectra Manager Program version 1.53 (JASCO Corporation, Japan) as the average of two repetitions with a data bin of 1 nm and a scan speed of 50 nm/min. All spectra had the background obtained with buffer alone and subtracted from them.

Thioflavin T (ThT) spectroscopy assay: 2 μL of 1 mM ThT solution were added to 198 μL of peptide solution (1 mg/mL). The fluorescence was measured immediately on a 96-well (BD Biosciences) with a SpectraMax M2 plate reader (444 nm excitation and 485 nm emission filters).

Thioflavin T (ThT) fluorescence microscopy assay: 0.75 μL of 100 μM ThT solution were added to 14.25 μL of protein sample. The sample was deposited on a clean glass slide and covered with a cover slip. The sample was imaged immediately with the Axio Imager fluorescence microscope (Zeiss, Germany). ThT-induced fluorescent fibers were observed with

blue (430-465 nm), cyan (465-500 nm) and green (500-550 nm) filters, with the strongest fluorescence observed with the cyan filter.

SI Figures:

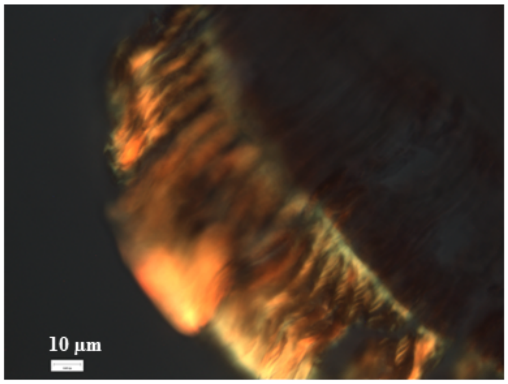

Figure S1| Polarized light microscopy image of wild-type (WT) enamel ultrastructure section stained with Congo Red obtained from incisor corresponding to early maturation stage.

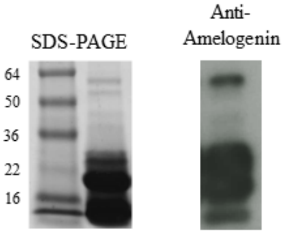

Figure S2| Western blot analysis of porcine enamel extract by SDS-PAGE (left) and SDS-PAGE stained with amelogenin specific antibodies (right). Porcine enamel extract bands stain positive with amelogenin antibodies.

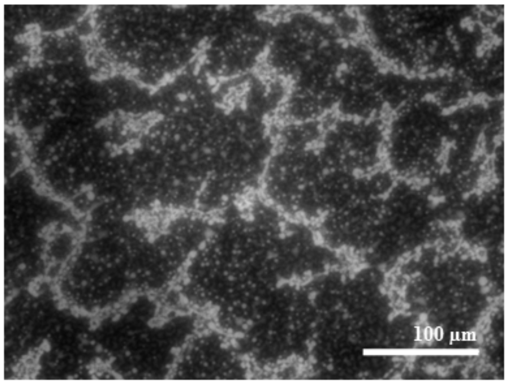

Figure S3| Porcine enamel extract stains positive for amyloids with Thioflavin T.

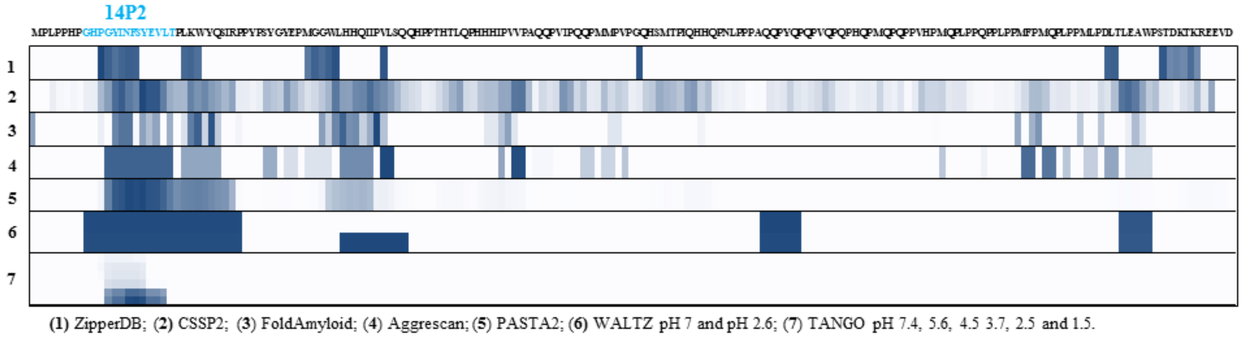

Figure S4| Seven sequence analysis methods predict amyloid propensity for several regions in amelogenin at different pHs: (1) ZipperDB, (2) CSSP2, (3) FoldAmyloid, (4) Aggrescan, (5) PASTA2, (6) WALTZ pH 7 and 2.6 respectively, (7) TANGO pH 7.4, 5.6, 4.5, 3.7, 2.5 and 1.5 respectively. All analysis methods used predict a high amyloid aggregation score for the 14P2 region.

	**2.29 mM NaH_2PO_4 pH = 4.5**	**2.29 mM NaH_2PO_4 pH = 6.5**	**3.36 mM $CaCl_2$ pH = 4.5**	**3.36 mM $CaCl_2$ pH = 6.5**	**Ca+P pH = 4.5**
$\Delta G(K_BT)$	17.36 ± 1.55	14.08 ± 2.25	15.33 ± 0.62	10.70 ± 1.62	13.40 ± 0.40

Table S1| Binding energy between 14P2 molecules in different environments measured by dynamic force spectroscopy (DFS).

14P2 deleted

MPLPPHP ■ ■ ■ ■ ■ ■ ■ ■ ■ ■ ■ ■ ■ PLKWYQSIRPPYPSYGYEPMGGWLHHQIIPVLSQQHPPTHTLQPHHHIPVVPAQQPV

IPQQPMMPVPGQHSMTPIQHHQPNLPPPAQQPYQPQPVQPQPHQPMQPQPPVHPMQPLPPQPPLPPMFPMQPLPPMLPDL

TLEAWPSTDKTKREEVD

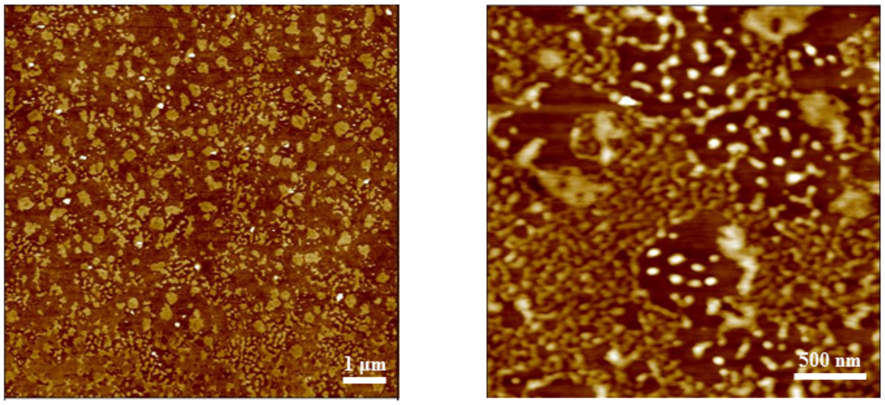

Figure S5| Amino acid sequence of 14P2-del-rH174 (top) and AFM images of its ill-defined self-assembly due to the lack of 14P2 region.

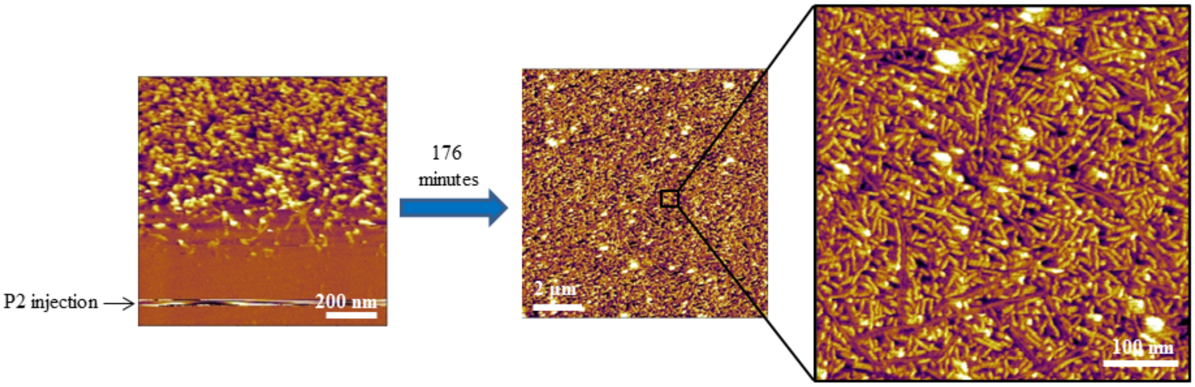

Figure S6| *In situ* AFM of 14P2 assembly in the presence of calcium and phosphate ions on mica at high peptide concentration (1 mg/ml). In this case, 14P2 forms aggregates that elongate into fibers with time (around 176 minutes).

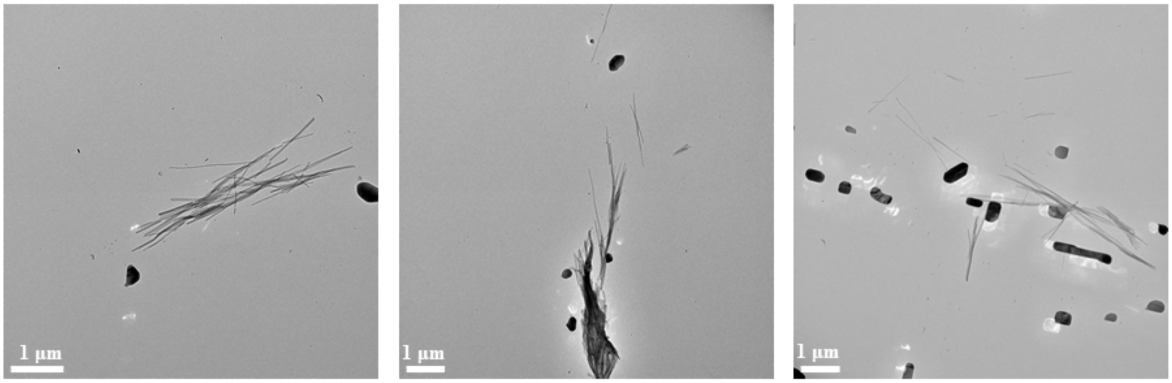

Figure S7| Representative unstained TEM images of 14P2 peptide in the presence of calcium and phosphate ions.

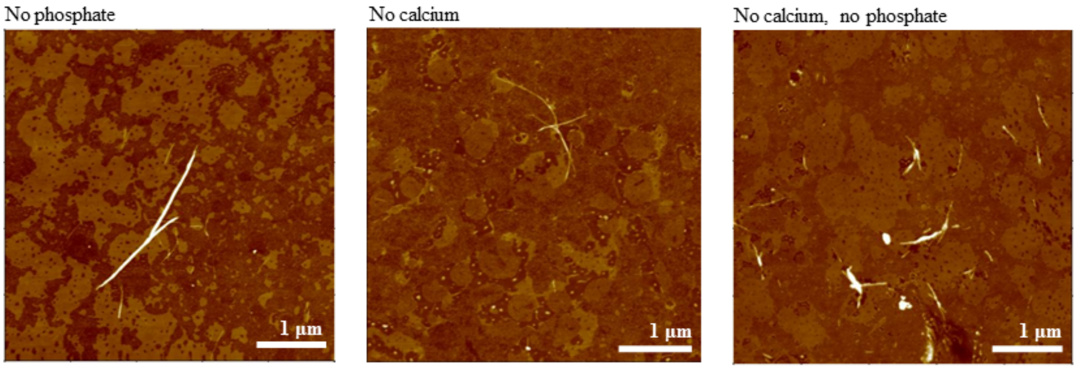

Figure S8| AFM images of 14P2 with no added phosphate (left), no added calcium (middle) and no added calcium and phosphate (right). In all cases, very few ribbons were observed.

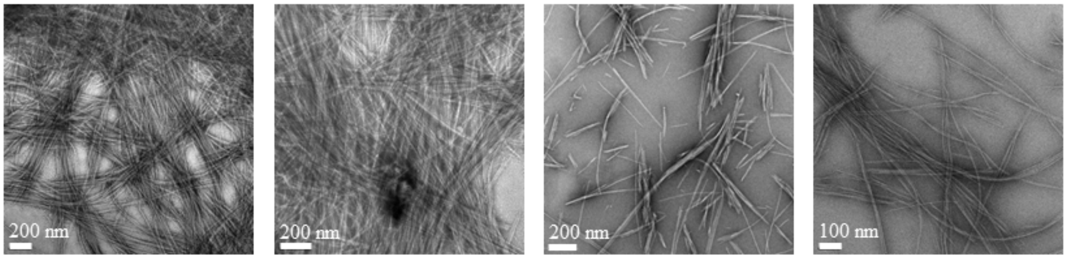

Figure S9| TEM images of negatively stained $_{p}$14P2 samples in the presence of calcium and phosphate ions at pH 4.5.

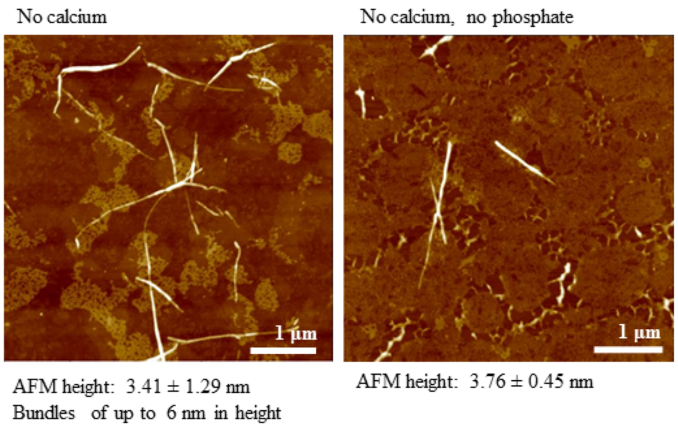

Figure S10| AFM images of $_{p}$14P2 with no added calcium (left) and no added calcium and phosphate (right). Very few ribbons were observed when calcium was not present in solution.

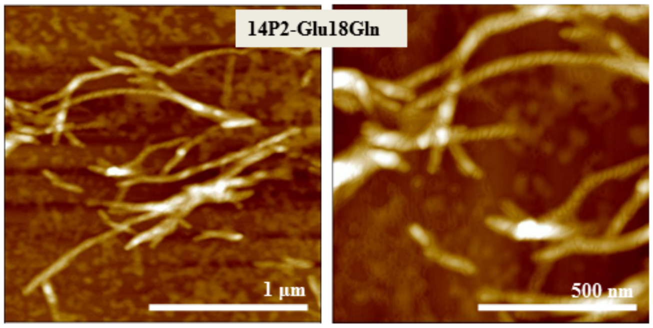

Figure S11| AFM image of modified 14P2 peptide, 14P2-Glu18Gln showing helical fibers.

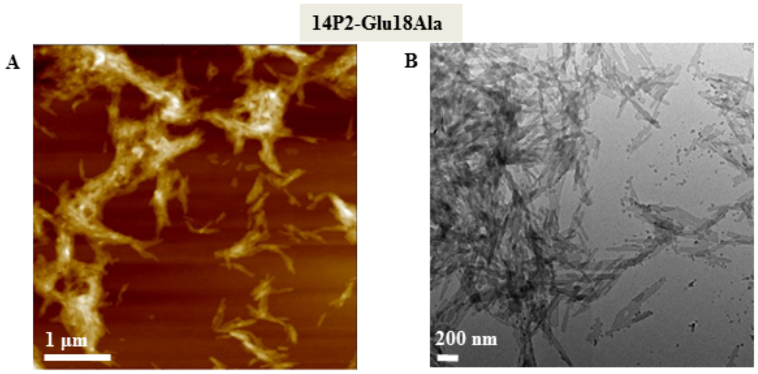

Figure S12| Self-assembly of modified 14P2 peptide, 14P2-Glu18Ala, into nanosheets by (A) AFM and (B) TEM.

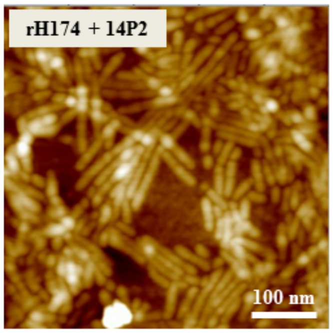

Figure S13| AFM of ribbons formed when rH174 and 14P2 were co-assembled at a ratio of (10:1). Nanoribbons were observed within 3-5 days of incubation. 14P2 can act as a seed to speed up the assembly of rH174.

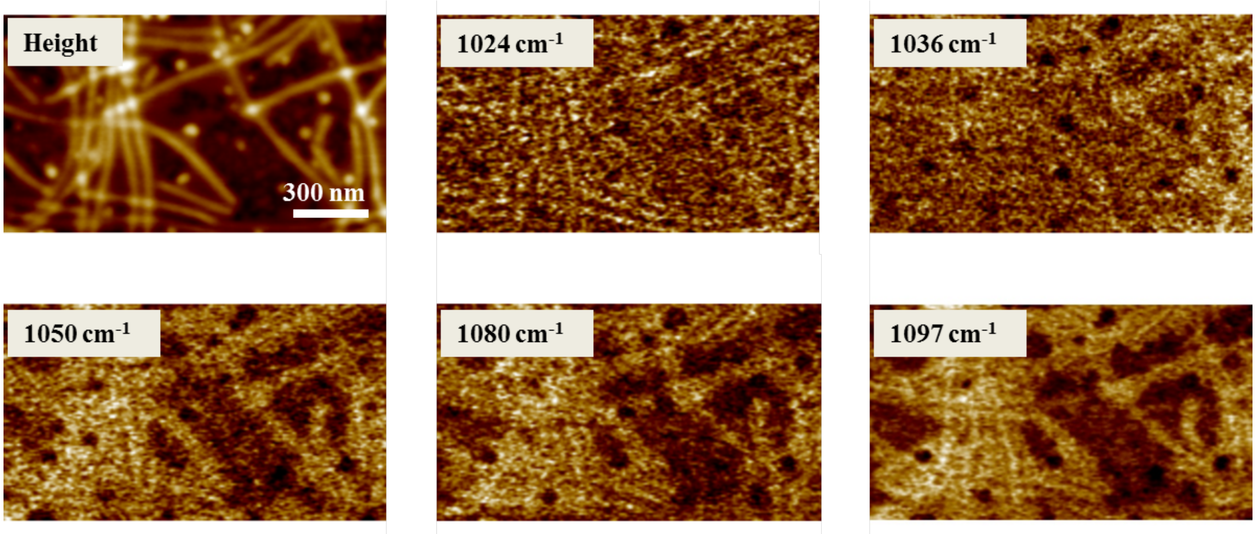

Figure S14| Topography and AFM-based s-SNOM measurements of rH174-(+9) at different wavenumbers in the infrared. Strong on-resonance absorption is observed at 1097 cm^{-1}, indicating the distribution of phosphates along the protein nanoribbons.

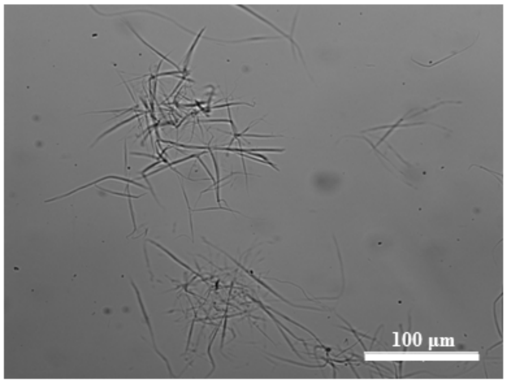

Figure S15| Optical microscope image (20x magnification) of rH174-(+9) nanoribbon bundles assembled in magnesium and phosphate ions at pH 4.5.

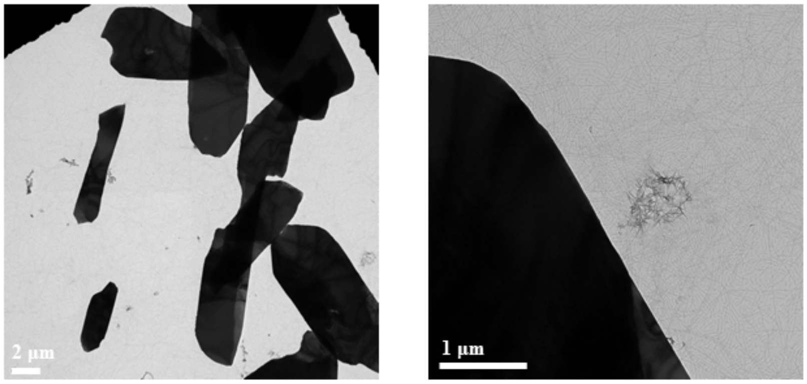

Figure S16| TEM images of hydroxyapatite precipitates in rH174-(+9) samples at pH 6.5.

References:

1. Chen E*, et al.* The small bovine amelogenin LRAP fails to rescue the amelogenin null phenotype. *Calcif. Tissue Int.* **73(5)**, 487-495 (2003).
